# Parental Corrections

**Published:** 1880-10

**Authors:** 


### PARENTAL CORRECTIONS.

THAT man commits a crime, and so does the woman who will send a child to bed with a wounded spirit, or who shall allow any vindictiveness of feeling to exist in consequence of any thing the child may have done. Sharp-pointed memories have often driven men mad; multitudes are there who are more dead than alive, from the ailings of the mind, which is wasting itself away in vain remorses for the irrevocable past. The fault of most parents is over-harsh reproofs of their children; reproofs that are hasty, unproportioned to the offense, and hence as to one's own child, helpless and unresisting, are a cruelty as well as an injustice. Thrice happy is that parent who has no child in the grave which can be wished back, only if for a brief space, so as to afford some opportunity for repairing some unmerited unkindness toward the dead darling. Parents have been many times urged in these pages to make persistent efforts to arrange two things in domestic intercourse, and to spare no pains and no amount of moral courage and determination, in order that they should be brought about. It may require a thousand efforts, and there may be a thousand failures, as discouraging as they are sad; still let the high resolve go out, "it shall be done!" and the prickling of many a thorn will be spared in after years and in old age. The two points to be daily aimed at are:

First. Let the family table be always a meeting-place of pleasantness and affection and peace, and for the exhibition of all the sweeter feelings of domestic life.

Second. Let every child be sent to bed with kisses of affection, especially those under ten years of age.

All that is on this globe could not hire me to be put in the place of either the father or the mother in the following narration of the former editor of a monthly of deserved repute in its time. The occurrence took place in Boston, about the year 1850, and every detail is minutely and literally true:

"A few weeks before, L. B. H—— wrote to me that he had buried his eldest son, a fine, manly little fellow of eight years of age, who had never known a day's illness until that which finally removed him hence, to be here no more. His death occurred under circumstances which were peculiarly painful to his parents. A younger brother, a delicate, sickly child from its birth, the next in age to him, had been down for nearly a fortnight with an epidemic fever. In consequence of the nature of the disease, every precaution had been adopted that prudence suggested to guard the other members of the family against it. But of this one, the father's eldest, he said he had little to fear, so rugged was he and so generally healthy. Still, however, he kept a vigilant eye upon him, and especially forbade his going into the pools and docks near his school, which it was his custom sometimes to visit; for he was but a boy, and 'boys will be boys,' and we ought more frequently to think that it is their nature to be. Of all unnatural things, a reproach almost to childish frankness and innocence, save me from a 'boy-man!' But to the story.

"One evening this unhappy father came home, wearied with a long day's hard labor, and vexed at some little disappointments which had soured his naturally kind disposition, and rendered him peculiarly susceptible to the smallest annoyance. While he was sitting by the fire, in this unhappy mood of mind, his wife entered the apartment, and said:

"'Henry has just come in, and he is a perfect fright! He is covered from head to foot with dock-mud, and is as wet as a drowned rat!'

"'Where is he?' asked the father sternly.

"'He is shivering over the kitchen-fire. He was afraid to come up here when the girl told him you had come home.'

"'Tell Jane to tell him to come here this instant!' was the brief reply to this information

"Presently the poor boy entered, half perished with affright and cold. His father glanced at his sad plight, reproached him bitterly with his disobedience, spoke of the punishment which awaited him in the morning, as the penalty for his offense, and in a harsh voice concluded with:

"'Now, sir, go to your bed!'

"'But, father,' said the little fellow, 'I want to tell you——'

"'Not a word, sir; *go to bed!*'

"'I only wanted to say, father, that——'

"With a peremptory stamp, an imperative wave of his hand toward the door, and a frown upon his brow, did that father without other speech, again close the door of explanation and expostulation.

"When the boy had gone supperless and sad to his bed, the father sat restless and uneasy while supper was being prepared, and at tea-table ate but little. His wife saw the real cause, or the additional cause of his emotion, and interposed the remark:

"'I think, my dear, you ought at least to have heard what Henry had to say. My heart ached for him when he turned away with his eyes full of tears. Henry is a good boy, after all, if he does sometimes do wrong. He is a tender-hearted, affectionate boy. He always was.'

"And therewithal the water stood in the eyes of that forgiving mother, even as it stood in the eyes of Mercy, in 'the house of the Interpreter,' as recorded by Bunyan.

"After tea the evening paper was taken up; but there was no news and nothing of interest for that father in the journal of that evening. He sat for some time in an evidently painful reverie, and then rose and repaired to his bed-chamber. As he passed the bedroom where his little boy slept, he thought he would look in upon him before retiring to rest. He crept to his low cot and bent over him. A big tear had stolen down the boy's cheek and rested upon it, but he was sleeping calmly and sweetly. The father deeply regretted his harshness as he gazed upon his son, but he felt also the 'sense of duty;' yet in the night, talking the matter over with the lad's mother, he resolved and promised, instead of punishing, as he had threatened, to make amends to the boy's aggrieved spirit in the morning for the manner in which he had repelled all explanation of his offense.

"But that morning never came to the poor child in health. He awoke the next morning with a raging fever on his brain, and wild with delirium. In forty-eight hours he was in his shroud. He knew neither his father nor his mother, when they were first called to his bedside, nor at any moment afterward. Waiting, watching for one token of recognition, hour after hour, in speechless agony, did that unhappy father bend over the couch of his dying son. Once, indeed, he thought he saw a smile of recognition light up his dying eye, and he leaned eagerly forward, for he would have given worlds to have whispered one kind word in his ear and have been answered; but that gleam of apparent intelligence passed quickly away, and was succeeded by the cold, unmeaning glare and the wild tossing of the fevered limbs, which lasted until death came to his relief.

"Two days afterward the undertaker came with the little coffin, and his son, a playmate of the deceased boy, bringing the low stools on which it was to stand in the entry-hall.

"'I was with Henry,' said the lad, 'when he got into the water. We were playing down at the Long Wharf, Henry and Frank Mumford and I; and the tide was out very low, and there was a beam run out from the wharf, and Charles got out on it to get a fish-line and hook that hung over where the water was deep, and the first thing we saw he had slipped off and was struggling in the water! Henry threw off his cap and jumped clear from the wharf into the water, and after a great deal of hard work, got Charles out; and they waded up through the mud to where the wharf was not so wet and slippery, and then I helped them to climb up the side. Charles told Henry not to say any thing about it, for if he did his father would never let him go near the water again. Henry was very sorry, and all the way going home he kept saying:

"'What will father say when he sees me to-night? I wish we had not gone to the wharf!'

"'Dear, brave boy!' exclaimed the bereaved father; 'and this was the explanation which I so cruelly refused to hear! And hot and bitter tears rolled down his cheeks.

"Yes, that stern father now learned, and for the first time, that what he had treated with unwonted severity as a fault, was but the impulse of a generous nature, which, forgetful of self, had hazarded life for another. It was but the quick prompting of that manly spirit which he himself had always endeavored to graft upon his susceptible mind, and which, young as he was, had already manifested itself on more than one occasion.

"Let me close this story in the very words of that father, and let the lesson sink deep into the heart of every parent who shall peruse this sketch:

"'Every thing that I now see that ever belonged to him reminds me of my lost boy. Yesterday I found some rude pencil-sketches, which it was his delight to make for the amusement of his younger brother. To-day, in rummaging an old closet, I came across his boots, still covered with dock-mud, as when he last wore them. (You may think it strange, but that which is usually so unsightly an object is now "most precious to me.") And every morning and evening I pass the ground where my son's voice rang the merriest among his playmates.

"'All these things speak to me vividly of his active life, but I can not—though I have often tried—I can not recall any other expression on the dear boy's face than that mute, mournful one with which he turned from me on the night I so harshly repulsed him. . . . Then my heart bleeds afresh!

"'Oh! how careful should we all be that in our daily conduct toward those little beings sent us by a kind Providence, we are not laying up for ourselves the sources of many a future bitter tear! How cautious that, neither by inconsiderate nor cruel word or look, we unjustly grieve their generous feeling! And how guardedly ought we to weigh every action against its motive, lest, in a moment of excitement, we be led to mete out to the venial errors of the heart the punishment due only to willful crime!

"'Alas! perhaps few parents suspect how often the fierce rebuke, the sudden blow, is answered in their children by the tears, not of passion, not of physical or mental pain, but of a loving yet grieved or outraged nature!'"

But why in this sad case should the mother be called to weep tears of blood, and be considered a partaker of the father's fault? It was for the criminal want of judgment and consideration on her part. The father had come home wearied and discouraged in connection with the business of the day, was sitting by the fire in a moody state of mind, and the mother bursts in upon him with the announcement of the boy's condition, without acquainting herself with the circumstances, and without uttering one word of extenuation, but presenting the case to the father's mind in the strongest terms of aggravation. No wonder, under all the circumstances, the husband should have fired up, and that he should have been driven on like one unpossessed of himself. Had the mother possessed but a small share of observation, and even a less amount of common-sense, she would herself have inquired into all the circumstances of the case, and began the history by extolling the nobleness of their son; then it would have had a calming, compensating effect on the father's mind; it would have been drawn away from business, and would have nestled itself lovingly amid the darling ones around him.

Even if there had been no extenuating circumstances, she ought to have had wit enough to have respected the humor of her husband; she ought to have seen in a moment that something had gone wrong with him, and should have studiously kept from saying or doing any thing which could by any possibility have roused him into a tempest of uncontrollable passion. There are many other just such thoughtless, hare-brained women, who deserve neither the name of mother nor wife, who seem to glory in dashing at their husbands the instant they open the door, on their return from a hard day's toil, of body or of mind, and with amazing volubility, pour out the mishaps, vexations, and misfortunes of the day, and in a way, too, as if the husband was wholly to blame, although he may not have had the slightest connection with them, in the most remote manner possible.

Another inexcusable folly was in the father threatening to punish the child next day; leaving the little fellow's mind to exaggerate it in his fears, and be a living torture until the end came. Not long ago, we read an account of an editor who sent his little son to an up-stairs room, and had the door locked, with the threat that he would be flogged at the end of a certain number of hours. True to his word, he went to the door at the appointed time, and in the unlocking of it the child was so alarmed, that he ran to the window, jumped out, and broke his neck. It is the limit of folly and the refinement of cruelty to threaten punishment to a child for a thing done. If punishment is merited, it should be inflicted and then dismissed; yet there are parents not a few who seem to have a malignant pleasure, after children have been reproved or otherwise punished for a specific fault, in reminding them of it on every possible occasion for months afterward; the certain effect of which is to induce a kind of desperation in the mind of the child and a "don't care" feeling, which can not fail to have a most unfortunate influence on that child's character for all its life thereafter.

Let parents, then, who would avoid an old age of agony, in connection with harshness, injustice, and even cruelty to their children, remember never to punish or even threaten a child under the influence of a passionate state of the mind, because the morrow may bring death, and no compensation can be ever made.

There is a physiological view to be taken of this case, which may be communicated with profit. Even if the child had been ever so much to blame, he should have been tenderly dealt with as to the present. His mind and body had been most intensely exercised, and the reäction had left the whole system in a state of complete exhaustion. In addition, the body was chilled. He should have been cleansed and re-dressed with all a mother's affection; a warm supper and some hot drink should have been given him, and he should have been put to sleep tenderly, in a warm bed. But instead of all this, he was cold, wet, hungry, "shivering," sent to bed, his feelings "hurt" to an extent which words can not express. We almost feel as if the father of the unfortunate boy was entitled to the designation of "savage," and his wife, a poor, hasty, weak-minded nonentity—worse than no wife at all.

---

SLEEPING-ROOMS should never be papered, and, most of all, with paper having any green color, whatever paper makers may say to the contrary.

